# The Effect of Exogenous Ketones on Signs and Symptoms of Schizophrenia Spectrum and Bipolar Disorders: Study Protocol for a Triple-Blind, Randomized, Controlled Crossover Pilot Study

**DOI:** 10.1016/j.cdnut.2025.107480

**Published:** 2025-05-24

**Authors:** Daphne AM Dielemans, Yagmur Yurtkap, Marieke van der Pluijm, Maarten R Soeters, Bob Oranje, Dirk JA Smit, Tim Ziermans, Mirjam J van Tricht, Sriram Muthukumar, Shalini Prasad, Romée L van der Mieden van Opmeer, Eline Dekeyster, Astrid M Kamperman, Jason RB Dyck, Bram-Sieben Rosema, Rocco Hoekstra, Ralph W Kupka, Lieuwe de Haan, Nico JM van Beveren, Karin Huizer

**Affiliations:** 1 Parnassia Psychiatric Institute, The Hague, the Netherlands; 2 Department of Psychiatry, Amsterdam University Medical Center, Amsterdam, the Netherlands; 3 Department of Endocrinology and Metabolism Amsterdam University Medical Center, Amsterdam, the Netherlands; 4 Center for Neuropsychiatric Schizophrenia Research, Academic Hospital Glostrup, Glostrup, Denmark; 5 Dutch Autism & ADHD Research Center, University of Amsterdam, Amsterdam, the Netherlands; 6 Department of Neuropsychiatry, GGZ Centraal, Laren, the Netherlands; 7 Enlisense LLC, Allen, TX, United States; 8 Department of Bioengineering, University of Texas at Dallas, Richardson, TX, United States; 9 Department of Cognitive Psychology, Leiden University, Leiden, the Netherlands; 10 Epidemiological and Social Psychiatric Research Institute, Department of Psychiatry, Erasmus Medical Center, Rotterdam, the Netherlands; 11 Cardiovascular Research Centre, Department of Pediatrics, University of Alberta, Edmonton, Alberta, Canada; 12 Department of Psychiatry, University Medical Center Groningen, Groningen, the Netherlands; 13 Department of Pathology, Erasmus Medical Center, Rotterdam, the Netherlands

**Keywords:** exogenous ketones, ketone ester, schizophrenia, bipolar disorder, ketosis, treatment, prepulse inhibition

## Abstract

**Background:**

Inflammation, oxidative stress, and bioenergetic dysfunction are proposed underlying mechanisms of schizophrenia spectrum disorders (SSDs) and bipolar disorders (BDs), contributing to the largely untreated cognitive and negative symptoms in these conditions. Ketone bodies may offer a therapeutic option for these symptoms through their positive effects on the aforementioned mechanisms. Exogenous ketones like ketone esters (KEs) provide a means to quickly induce ketosis without dietary restrictions, though their effects on SSD and BD have not yet been investigated.

**Objectives:**

This ongoing triple-blind, randomized controlled crossover trial investigates the effects of a single ingestion of KE on signs and symptoms of SSD and BD.

**Methods:**

A total of 24 patients (12 SSD and 12 BD) receiving inpatient care at Amsterdam University Medical Center (UMC) will be included in the study. Patients will ingest a single dose of KE ((R)-3-hydroxybutyl-(R)-3-hydroxybutyrate deltaG Ketones (dGK) and an isocaloric carbohydrate control with a washout period of 3 days between drinks. The primary outcome is the change in prepulse inhibition of the startle reflex induced by dGK ingestion compared with control. Secondary outcomes include resting-state electroencephalography, P3B amplitude, cognitive performance, and metabolic, immune, oxidative stress, and circadian rhythm parameters. Feasibility and potential side effects will also be assessed.

**Results:**

N/A (study protocol).

**Conclusions:**

Our current study will offer valuable preliminary data on the effects of KE in patients with SSD and BD. It can provide the foundation for future research into the therapeutic potential of KE in alleviating symptoms and improving functional outcomes in these disorders.

This trial was registered at www.clinicaltrials.gov as NCT06426134.

## Introduction

Schizophrenia spectrum disorders (SSDs) and bipolar disorders (BDs) are severe mental disorders associated with a high disease burden and increased mortality [1]. SSD is characterized by positive symptoms (e.g., hallucinations, delusions), negative symptoms (e.g., lack of motivation and social withdrawal), and cognitive symptoms (e.g., difficulties with executive functions and working memory). BD involves (hypo)manic episodes often alternating with depressive episodes. Antipsychotic medications, which mainly inhibit the dopamine D_2_ receptor, are an important form of treatment for both disorders. The effectiveness of dopamine receptor antagonists in alleviating positive symptoms of SSD has been pivotal in the development of the dopamine hypothesis [2].

Although antipsychotics are effective in reducing positive and manic symptoms, they fail to address cognitive [[3], [4], [5], [6], [7], [8]] or negative [9,10] symptoms, which significantly contribute to the chronic disease burden of SSD or BD. Additionally, antipsychotics can generate secondary negative symptoms, such as affect flattening and lack of motivation, as well as cognitive dysfunction at higher doses, thereby exacerbating the burden of negative and cognitive symptoms [11,12]. Besides improving cognition and negative symptoms, another unmet treatment need concerns modification of etiopathophysiological mechanisms. Mechanisms such as glutamatergic dysregulation, oxidative stress, and inflammation are thought to play a central role in SSD and BD [[13], [14], [15]]. The glutamate hypothesis posits that disruptions in glutamatergic signaling, particularly involving N-methyl-D-aspartate receptors, contribute to the symptoms of SSD and BD [14,16]. Oxidative stress refers to an imbalance between the production and neutralization of reactive oxygen species (ROS). Excessive ROS can induce oxidative damage to cellular macromolecules, including lipids, proteins, and DNA, resulting in impaired neuronal function and synaptic dysfunction [13]. Oxidative stress and inflammation are interconnected mechanisms often described together in the context of SSD and BD. Recent evidence suggests that disruptions in glucose metabolism may underlie these disorders [[17], [18], [19]]. According to the bioenergetic dysfunction hypothesis, the capacity of the brain to efficiently utilize glucose for ATP production may be impaired. Reduced glycolytic enzyme activity and mitochondrial dysfunction have been suggested as underlying mechanisms [19]. This disrupted energy homeostasis is thought to impair neuronal communication, contributing to cognitive deficits and negative symptoms [18,19].

Nutritional ketosis (NK)—an increase in blood ketone levels through nutritional interventions—may offer clinical benefits for SSD or BD by improving bioenergetic deficits [20,21]. NK can be induced through fasting, consumption of medium-chain triglycerides (MCT), ingestion of exogenous ketones, or adherence to a ketogenic diet (KD), which is characterized by high-fat and low-carbohydrate intake [[22], [23], [24]]. During fasting or a KD, ketone bodies [primarily β-hydroxybutyrate (BHB) and acetoacetate (AcAc)] are produced via the β-oxidation of fatty acids in the liver (ketogenesis) and are released into the blood stream afterward. It commonly takes several days to a few weeks to fully adjust to a KD. When exogenous ketones, such as a ketone ester (KE) are ingested, ketone bodies become immediately available as an energy source upon resorption. The brain can effectively use ketone bodies as an alternative energy source: ketone bodies are efficiently transported across the blood–brain barrier [25,26] and metabolized in neural mitochondria for ATP production [21]. Furthermore, KE intake in nonfasting mice resulted in increased brain ketone uptake and reduced glucose metabolism [27], suggesting that ketones are preferentially utilized when available. NK is also thought to improve neuronal energy metabolism by stimulating mitochondrial biogenesis, which enhances mitochondrial function and ATP production [22,23]. When ketone bodies are available, the brain may, therefore, bypass energy metabolism defects [28] observed in patients with SSD and BD.

The effects of NK on the brain extend beyond providing an alternative energy source to glucose for neuronal mitochondria. Ketone bodies can mitigate oxidative stress and inflammation, by increasing the NAD+/NADH ratio in mitochondria, thus decreasing ROS production and enhancing ATP synthesis [29]. Additionally, the antioxidant properties of ketone bodies may be effective in reducing neuronal cell death [30]. Given the close relationship between oxidative stress and inflammation, NK may indirectly modulate the immune responses by reducing oxidative stress. However, ketone bodies can directly target the inflammatory system through various pathways [23,31]. Indeed, the KD has demonstrated neuroprotective effects by inhibiting inflammation induced by macrophages and microglia in the central nervous system [32]. Finally, ketone bodies also suppress neuro-inflammation in an acute inflammation mouse model by preventing elevated transcription of proinflammatory cytokines involved in SSD and BD (IL-6, TNF-α, and IL-1β) [20]. Furthermore, ketone bodies influence glutamate metabolism by increasing glutathione levels [22] and can reduce glutamate-induced excitotoxicity [33,34]. In summary, previous studies have highlighted multiple mechanisms through which NK may exert its effects, including its impact on mitochondrial energy production, oxidative stress, inflammation, and neurotransmitter levels. These mechanisms overlap with key pathophysiological processes involved in SSD and BD, underscoring the potential of NK as a therapeutic approach for these disorders.

In clinical research, a KD has demonstrated potential for improving cognitive function [[35], [36], [37]], modulating the immune system [38,39], and addressing metabolic syndrome [40,41] in various nonpsychiatric contexts. Some findings indicate improved sleep quality following a KD, possibly by influencing the circadian rhythm through ketone bodies [34]. Although the KD is an evidence-based treatment method for treatment-resistant epilepsy [42,43], research into its efficacy in psychiatric diseases like SSD or BD is still in its early stages. Small and uncontrolled trials suggest beneficial effects on metabolic health, functional outcomes, psychotic symptoms in SSD, and mood stability in BD [[44], [45], [46], [47], [48], [49]], and an RCT investigating the effects of the KD in SSD is currently ongoing [50]. Nonetheless, a knowledge gap remains regarding the potential positive effects of NK on cognitive and negative symptoms in SSD and BD. Data from SSD animal studies support positive effects of ketones on deficits in sensorimotor gating (a process essential for adequate cognitive functioning), as measured by prepulse inhibition (PPI) of the startle reflex (PPI) [51,52]. A single dose of exogenous ketones could reverse PPI deficits in this animal model [52]. Because PPI is often disrupted in SSD and BD [53,54], this is a promising finding.

Although a KD may be a realistic option to achieve ketosis for some, its strict carbohydrate restriction is generally considered a major hurdle often leading to low or moderate adherence [55,56]. When acute psychiatric episodes or negative symptoms like loss of motivation are present, maintaining such a diet may be particularly difficult. The ingestion of KE may induce acute and potent ketosis without the need for a challenging diet [27,57]. The KE (R)-3-hydroxybutyl (R)-3-hydroxybutyrate [deltaG Ketones (dGK)] has been available as a food product for consumers for several years. Research indicates its efficacy in improving outcomes in conditions such as Alzheimer’s disease, Parkinson’s disease, and type 2 diabetes, as well as in enhancing athletic performance [[58], [59], [60]]. To the best of our knowledge, no published studies to date have evaluated the efficacy of KE supplementation as a standalone intervention without dietary restrictions in individuals with SSD or BD.

Here, we present a research protocol for a pilot study currently conducted at Amsterdam University Medical Center (UMC), the Netherlands, which investigates the effects of KE ingestion in patients with SSD or BD. Our current study is part of a broader research initiative aimed at identifying novel therapeutic strategies for addressing negative and cognitive symptoms in SSD and BD.

## Methods

### Ethics statement

The study has been approved by the Amsterdam UMC Institutional Review Board (IRB/Medical Research Ethics Committee, AUMC) under the number 2024.0100, in accordance with the Medical Research Involving Human Subjects Act (WMO) which is based on the Nuremberg Code, the Declaration of Helsinki (version October 2013) and the International Council For Harmonization—Good Clinical Practice guideline. The study is conducted in accordance with the local legislation and institutional requirements. The participants provided their written informed consent to participate in this study.

### Aims and objectives

The aim of this pilot study is to investigate the effects of a single dose of dGK on signs and symptoms of SSD or BD and to explore possible downstream mechanisms of action. The primary outcome measure is the change in PPI following dGK ingestion compared with an isocaloric carbohydrate control drink in the same patient with SSD or BD. Secondary outcomes include resting-state electroencephalography (EEG), P3B amplitude, cognitive performance, and patient-reported measures on mood, energy levels, and focus. Additionally, immune, oxidative stress, metabolic and circadian rhythm parameters are assessed. We will also evaluate the feasibility of dGK ingestion by examining its palatability and potential side effects in this patient group. This pilot study is intended as a lead-up for a future larger trial with a longer KE administration and follow-up.

### Trial design and setting

**FIGURE 1:**
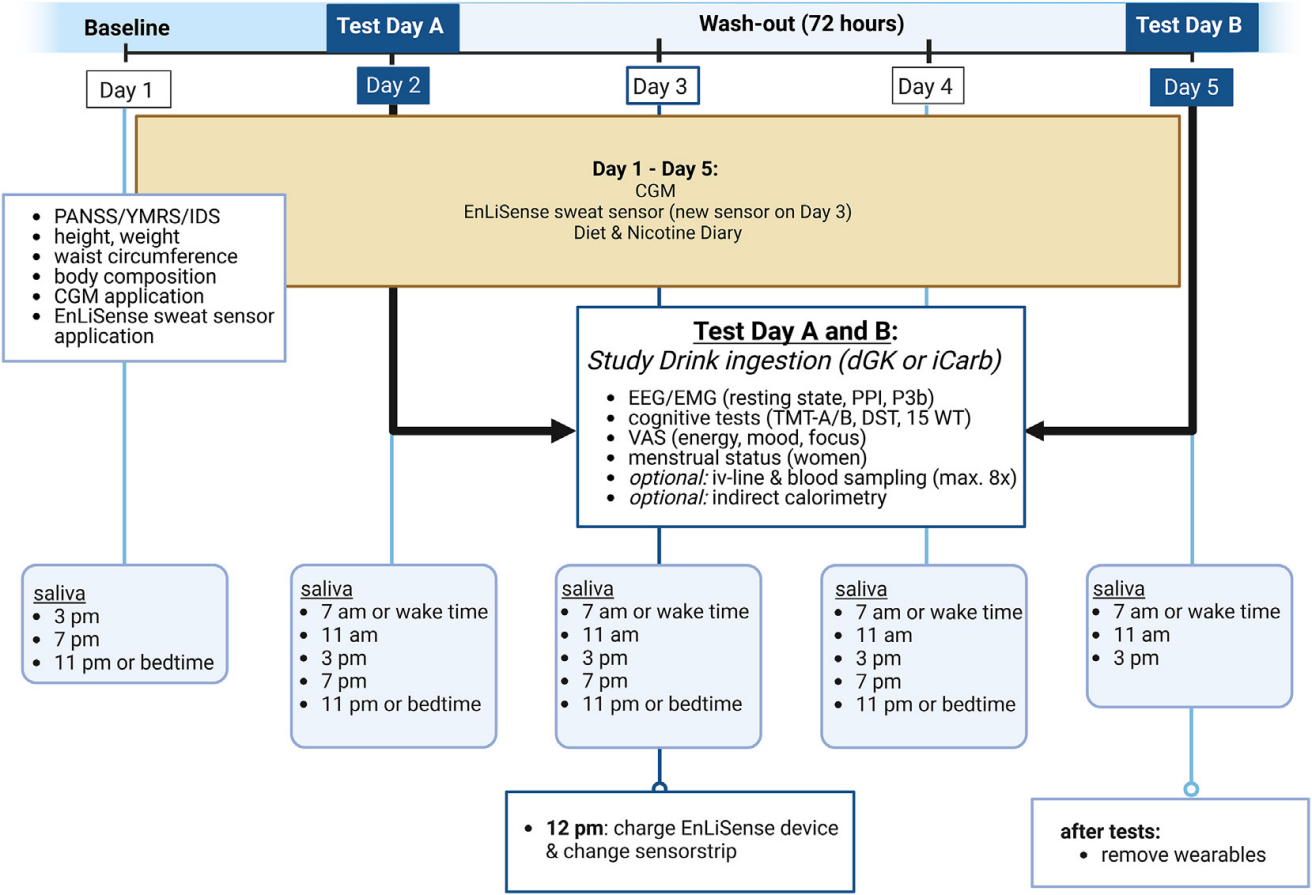
Overview of measurements during study participation. Schematic overview of the 5-day study protocol. On day 1, baseline assessments include psychiatric symptom scales [Positive and Negative Syndrome Scale (PANSS), Young Mania Rating Scale (YMRS), Inventory of Depressive Symptomatology (IDS-C)], anthropometric measures, and the application of continuous glucose monitors (CGMs) and sweat sensors. On days 2 and 5 (test days A and B), participants ingest either the ketone ester (deltaG, dGK) or an isocaloric carbohydrate control drink (iCarb). These test days include neuroelectrophysiological recordings [resting state, prepulse inhibition (PPI), and the P3b event-related potential], cognitive tests [Trail-Making Test A/B (TMT-A/B), Digit Span Test (DST), and the 15-Word Test (15 WT)], and visual analog scales (VAS). Optional procedures include intravenous blood sampling (≤8 times) and indirect calorimetry. EEG, electroencephalography; EMG, electromyography.

This pilot study is designed as a triple-blind, randomized controlled crossover trial. An isocaloric carbohydrate control is used to account for the potential effects of caloric intake on outcome measures and to control for any placebo effects. Both study drinks are generally considered to have an unpalatable taste and texture, aiding in blinding. The total study duration is 5 days, consisting of baseline measurements, 2 test days, and several continuous assessments. Participants ingest the KE or control drink orally on day 2 of the study, as determined by the randomization procedure. After a 72-h washout period, participants switch to the alternate condition on day 5. An overview of the scheduled measurements is provided in Figure 1, with a more comprehensive description presented in a later section.

The study is conducted in an academic hospital setting (Amsterdam UMC, the Netherlands), specifically within the early-episode psychosis ward and acute psychiatry ward of the department of psychiatry. As patients are already hospitalized for the treatment of their psychotic or bipolar episode, they can be closely monitored throughout the trial without requiring additional hospital visits. This minimizes the burden on patients and facilitates their participation in the study.

### Study population and eligibility criteria

#### Study population

The study population consists of patients with recent onset psychosis (defined as < 3 y since onset) because of SSD and patients with BD currently having a manic or depressive episode. There are no restrictions concerning sex or ethnicity. All patients receive treatment-as-usual based on Dutch guidelines, still have clinically evident disease symptoms but are sufficiently recovered to provide informed consent for study participation. The treating medical doctor or psychiatrist evaluates the patient's mental competence, upon which a researcher obtains informed consent after providing detailed information about the study's purpose, procedures, potential effects, and potential adverse events. The subject information provided to study participants explains the study, and also states the fact that both study drinks are generally considered unpalatable to aid blinding. Patients are given 1 wk to consider participation and receive reimbursement for participation. Participants receive a financial compensation of €200, either via bank transfer or as a gift card.

#### Inclusion criteria

The participant has a recent onset psychotic episode because of SSD, or a manic or depressive episode because of BD (both within 3 y of illness onset); age 18 y or older; receives treatment-as-usual, with the intervention provided alongside standard clinical care, typically consisting of antipsychotic and/or mood stabilizing medication, but not limited to these; treatment may be adjusted during participation in the study as clinically indicated; and the participant is competent to give informed consent.

#### Exclusion criteria

Substance use as cause of psychosis or mood episode; substance use (other than nicotine) in the week prior to study onset; intellectual disability (already established at admission to the hospital or clinically assessed); diabetes mellitus type 1 or type 2; metabolic disease impacting ketone metabolism, such as succinyl-CoA:3ketoacid CoA transferase deficiency, multiple acyl-CoA dehydrogenase deficiency; liver, kidney, and cardiovascular diseases; and the participant who is currently pregnant, attempting to become pregnant, or breastfeeding.

#### Withdrawal

Subjects can leave the study at any time for any reason if they wish to do so, without any consequences. The clinician or investigator can decide to withdraw a subject from the study for medical reasons. We expect a maximum drop-out percentage of 15% based on previous intervention studies within the same patient groups and setting.

### Intervention

#### Investigational product: dGK

The KE (R)-3-hydroxybutyl-(R)-3-hydroxybutyrate (dGK) was developed by TDeltaS Ltd., in collaboration with the University of Oxford. dGK is a United States Food and Drug Administration (“generally recognized as safe” approved food product. After ingestion, dGK is hydrolyzed in the small intestine, resulting in the production of BHB and 1,3-butanediol. Both are transported via the portal circulation to the liver, where 1,3-butanediol is metabolized into AcAc and BHB and released into the bloodstream. Currently, dGK has been available on the consumer market as a food product for several years, with extensive safety and toxicology studies conducted prior to its consumer release [61,62]. No serious adverse events have been reported. Infrequent nonserious side effects include mild gastrointestinal discomfort, dizziness, or headache for a short time [63].

The intervention consists of a nutritional drink containing 50 g of dGK (250 kcal). No correction for body weight is performed, because other factors outweigh body weight in the level of ketosis achieved [64]. The drink is cooled and diluted in water to a total volume of 150 mL to minimize the unpleasant flavor of dGK. After the ingestion of 50 g of dGK the time to reach the maximum peak blood concentration (Tmax) is between 1 and 3 h for BHB, and 1 and 4 h for AcAc [62].

#### Control product: isocaloric carbohydrate drink

A commercially available carbohydrate sports drink, Maurten Drink Mix 160, will serve as the isocaloric control condition. This drink contains maltodextrin and fructose as its primary ingredients. To match the caloric content of the dGK beverage, 63 grams of Maurten Drink Mix 160 (providing 250 kcal) is dissolved in 150 mL of water, resulting in a 150 mL control drink. Like dGK, Maurten Drink Mix 160 mixed into this volume of water is generally considered to have an unpalatable taste, which supports blinding, although the 2 drinks are not identical in flavor and consistency. A detailed overview of the nutrient composition of both the dGK and control drink is provided in Table 1.

**TABLE 1:** Nutritional composition of the 2 study drinks.

	Intervention product: DeltaG Ketones	Control product: Maurten Drink Mix 160
Main ingredient	(R)-3-hydroxybutyl-(R)-3-hydroxybutyrate (50 g)	Maltodextrin + Fructose (ratio 0.8:1)
Carbohydrates	0 g	63 g
Protein	0 g	0 g
Fat	Only from ester	0 g
Calories	250 kcal	250 kcal
Volume	150 mL	150 mL

The intervention product contains the ketone ester (R)-3-hydroxybutyl-(R)-3-hydroxybutyrate (DeltaG Ketones). The control drink contains isocaloric carbohydrates (maltodextrin and fructose). Both drinks have comparable volume and caloric content.

### Allocation and blinding

Patients are randomized to receive either the dGK or the isocaloric control placebo drink first, using an electronic case report form (eCRF) tool that is accessible via the internet (Castor Electronic Data Capture, castoredc.com). The randomization key is kept confidential, with an independent researcher responsible for preparing and labeling the drinks with codes unknown to patients and test administrators. This researcher is not involved in patient inclusion, test procedures, or data analysis. Both patients and test administrators remain blinded during test sessions. Data analysis is conducted by the study investigators in a blinded manner, with the randomization key revealed only after the analysis is completed, resulting in a triple-blind design.

### Measures

#### Baseline characteristics

Several baseline characteristics are collected to form a comprehensive profile of the participants. These include demographic information such as sex, age, and ethnicity. Physical measurements are taken: BMI (in kg/m^2^), waist circumference, and body composition analysis via a bio-impedance scale (Robi S11). Standard clinical laboratory tests, as part of routine care during admission, yield results for complete blood count, electrolytes, C-reactive protein, and liver, kidney, and thyroid function. Detailed data on medication use, relevant comorbidities, and other diagnoses are extracted from patient files. For female participants, menstrual phase will be recorded by asking participants about the first day of their previous menstruation, the average duration of their menstrual cycle, and any hormonal contraceptives used. Furthermore, specific clinical interviews are conducted to assess baseline psychiatric symptoms. The Positive and Negative Syndrome Scale (PANSS) [65] is administered to participants with a psychotic episode, the Young Mania Rating Scale (YMRS) [66] for participants with a manic episode, and the Inventory of Depressive Symptomatology (IDS-C) [67] for participants with a bipolar depression.

Participants continue their regular diet, but maintain a dietary diary throughout the study, recording their habitual intake of food and liquids to provide an overview of their dietary habits. Similarly, participants who use nicotine products keep a smoking diary for the duration of the study.

#### Primary outcome measure

PPI of the acoustic startle reflex reflects the brain’s ability to filter sensory information. When a soft prepulse sound precedes a loud, startle-eliciting noise, it inhibits the startle reflex, indicating healthy sensory filtering. A disruption in PPI, where the prepulse fails to inhibit the startle reflex, indicates impaired sensory filtering. PPI is considered as one of the of the key neurophysiological abnormalities in SSD and BD, contributing to symptomatic cognitive impairments [53,54,68,69]. Deficits in PPI are regarded as fundamental trait markers, present across different states of SSD [70]. Combined with the finding that a single dose of exogenous ketones could reverse PPI deficits in an SSD animal model [52], this was reason to select this measure as our primary outcome.

PPI of the startle reflex is assessed using an auditory paradigm with electromyography (EMG) of the orbicularis inferior muscle [71,72]. The PPI paradigm starts with a 1-min acclimation period of 70 dB white noise (used in the paradigm as background noise), followed by 3 blocks of trials featuring pulse alone (PA) and pre-pulse-pulse conditions superimposed on the background noise. The pulses consist of white noise sound bursts at 115 dB lasting 20 ms, with intertrial intervals randomly ranging between 10 and 20 s. Blocks 1 and 3 measure habituation through 8 PA trials each. Block 2 comprises 50 trials, including 10 PA trials identical to those in blocks 1 and 3. Additionally, block 2 includes 4 pre-pulse-pulse trials presented 10 times each: prepulse sound bursts (76 and 85 dB) delivered either 60 or 120 ms before the 115 dB pulse. All 50 trials in block 2 are presented in a pseudorandomized order (no 2 identical trial types are presented in direct succession). Pulses and prepulses are delivered using experiment control software (Presentation, version 24.1). Participants hear the background noise and stimuli through audiometric insert earphones (3M E-A-RTONE Insert Earphone, ER3A). Electrophysiological data are recorded with a calibrated EEG apparatus (EEGOsports system, ANT Neuro) using EEG caps (Waveguardoriginal). Participants will wear the EEG cap for the entire duration of the testing, which lasts around 2–3 h. These caps are equipped with droplead electrodes to measure EMG bipolarly via 2 electrodes placed below the right eye on the orbicularis inferior muscle. The PPI task will be administered 1 h after the intake of the dGK or placebo drink to allow sufficient time for absorption into the bloodstream, and achieve a concentration near Tmax [62]. The total duration of the PPI task is ∼20 min.

#### Secondary outcome measures

##### Resting-state EEG

Besides impaired PPI, resting-state EEG alterations are frequently reported in SSD and BD [[73], [74], [75], [76], [77]]. In this study, resting-state EEG recordings are conducted prior to drink ingestion and 30 min thereafter. Resting-state EEG will be recorded under first both eyes-open (gaze fixation on visual mark in front of participant) and second eyes-closed conditions, with each condition lasting 5 min. Participants are instructed to remain seated, maintain an upright posture, relax and minimize movement during the recording. We investigate power spectral differences, specifically increased slow oscillatory power (delta/theta) in the frontal regions.

##### P3B event-related potential

The P3B amplitude is a subcomponent of the P300 specific type of event-related potential, reflecting cognitive processes such as attention, working memory, and decision making. P3B amplitude is considered a state and trait marker in SSD [78]. Both SSD and BD are associated with reduced P3B amplitude and delayed latency [73,79], yet the potential effects of ketone bodies on P3B amplitude remain unexplored. P3B amplitude is often measured by providing a random visual or auditory oddball paradigm where participants respond to infrequent stimuli in a sequence of a frequently occurring (standard) stimulus [78]. During our P3B task, conducted ∼45 min after ingestion, 160 stimuli are presented in random order, with participants instructed to press a button upon detection of the “oddball” target (32 out of the 160 stimuli). The task is preceded by 10 practice trials. The standard (nontarget) stimulus consists of a 500 Hz pure tone, whereas the “oddball” target stimulus is 700 Hz, each with duration of 100 ms. Both stimuli are presented at an intensity of 80 dB, with a random interstimulus interval ranging from 1.2 to 1.5 s. Throughout the P3B task, brain activity is recorded using the same EEG equipment as mentioned above to capture neural responses (Fz, Cz, and Pz channels), following the presentation of these stimuli.

##### Cognitive outcome measures

Cognitive impairments in SSD and BD have been extensively studied, highlighting deficits in cognitive domains such as executive functioning and memory [80]. The effects of the study drink on cognitive functioning are assessed using 3 validated neuropsychological tests: the Trail-Making Tests, the Digit Span Test (DST), and the 15-Word Test (15 WT). Collectively, these tests assess attention, cognitive flexibility, executive functioning, working memory, and verbal memory. The Trail-Making Tests (TMT-A and TMT-B, 2007, Dutch translation; paper versions) measure visual-motor speed, attention, and cognitive flexibility, with TMT-B also reflecting executive functions such as task-shifting [81]. The DST, as part of the Wechsler Adult Intelligence Scale (fourth edition), evaluates auditory working memory capacity, both in terms of forward and backward digit recall, as well as cognitive control [82]. The 15 WT (a Dutch adaptation of the Rey Auditory Verbal Learning Test) assesses short- and long-term verbal memory through immediate recall, delayed recall, and recognition of previously presented words, providing a comprehensive measure of memory processes [83]. Parallel tests are used to minimize learning effects, which are otherwise compensated by the AB/BA crossover design of the study. Cognitive assessments take place ∼90 min after ingestion. Previous studies have reported improvements in working memory and executive functioning following a single administration of exogenous ketones, including both MCT and KE [36,84]. Based on this evidence, it is hypothesized that similar acute effects on cognitive performance may also be observable in the present clinical sample.

##### Patient-experienced and reported outcomes

Participants are asked to rate their energy level (from very exhausted to very energetic), mood (from very depressed to very happy), and ability to focus (from very poor to very well) using Visual Analog Scale at 2 timepoints: before and 2 h after ingestion of the nutritional drink, on both test days. Subjective improvements in mood and energy have been reported during ketogenic interventions, including among individuals with psychosis and bipolar disorder [47]. The patient-experienced outcomes are intended to provide exploratory insight into participants’ subjective experience of KE administration.

##### Immune, oxidative stress, and metabolic blood parameters

Participants may opt out of blood sampling. For those who consent, a venous cannula is inserted into a forearm vein at the start of each session to facilitate sequential blood draws without repeated venipunctures. Blood is collected at the following time points: before drink ingestion (baseline, T0), at 20-min intervals during the first hour after ingestion (T1–T3), and at 30-min intervals thereafter (T4–T7), with a maximum of 8 blood draws and 123 mL per session. At each timepoint, ∼15 mL of blood is drawn and distributed over 5 tubes: 1 for serum (5 mL), 1 for plasma isolation using heparin (5 mL), and 3 EDTA tubes, used, respectively, for ketone body determination (1 mL), acylcarnitine profiles (1 mL), and remaining plasma. For ketone body determination, the EDTA sample is immediately mixed (1:1 ratio) with perchloric acid and kept on ice to prevent conversion into and evaporation of acetone. In addition, a 3 mL Tempus Blood RNA tube is collected at 1 h (T3) after drink ingestion, for gene expression analysis using the metabolic and immune nCounter gene expression panels from Nanostring. Following collection, all blood samples are handled in accordance with standardized laboratory protocols. Processed supernatants and aliquots are subsequently stored at −80°C until further analysis.

Immune, oxidative stress, and metabolic markers are determined in serum, plasma, and deproteinized blood. Markers are chosen according to prior research findings. Inflammatory markers include IL-6 and IL-10, measured in plasma, and high-sensitivity C-reactive protein (and soluble intercellular adhesion molecule-1 in serum) [13,20,[85], [86], [87]]. Oxidative stress markers include glutathione S-transferase and superoxide dismutase and key enzymes in ROS regulation [88,89]. Metabolic markers include glucose, ketone bodies (BHB and AcAc), and acylcarnitine profile [90,91].

##### EnLiSense sweat sensor and continuous glucose monitor

Participants will wear a noninvasive sweat sensor (EnLiSense LLC) throughout the 5-day study period, enabling near-continuous multiplex monitoring of biomarkers in sweat. This study focuses on markers linked to circadian rhythms and inflammation. Studying circadian rhythms is challenging because of the invasive nature of frequent melatonin and cortisol sampling, the primary regulators of circadian rhythms [92]. The ability to continuously measure cortisol and melatonin levels could provide deeper insights into the circadian rhythms of patients with SSD and BD [93]. Moreover, evidence from in vivo studies suggests that the KD may influence circadian rhythms [94], and it has been hypothesized that improvements in the sleep-wake cycle could mediate the beneficial effects of ketone bodies in SSD and BD [95]. Furthermore, IL-6 and TNF-α will be measured, as these proinflammatory cytokines are often be elevated in patients with psychosis [13,87]. In an acute inflammation mouse model, KE prevents increased expression in the brain of proinflammatory cytokines involved in SSD (IL-6, TNF- α, and IL-1β) [20], suggesting KE could normalize levels of these cytokines in SSD/BD. Therefore, assessing whether these markers change following ketone drink consumption could provide valuable insights into its potential effects on inflammation.

Melatonin, cortisol, IL-6, and TNF-α are determined in saliva samples as a validation for the sweat sensor data (Ella Automated Immunoassay System, Bio-Techne). Saliva samples are collected at fixed time points daily: 07:00 (or when the participant wakes up), 11:00, 15:00, 21:00, and 23:00 (or before bedtime). Saliva is collected in Eppendorf tubes by passive drool method (BioSaliva collection aid). Saliva samples are stored at −80°C until further analysis.

Additionally, participants wear a continuous glucose monitor (CGM, Abbott Freestyle Libre 2) for the entire 5-day duration of the study to gain further into the effects of a KE on glucose levels [87,96].

##### Indirect calorimetry

Information on substrate utilization and energy metabolism is obtained through indirect calorimetry. Indirect calorimetry is an optional test procedure for study participants. This method involves measuring oxygen consumption and CO_2_ production using a ventilated hood system (Q-NRG, Cosmed), which allows for the automatic calculation of resting energy expenditure (REE) and the respiratory quotient (RQ). REE reflects the energy metabolism of the participant at rest. The RQ (the ratio of CO_2_ exhaled to O_2_ consumed) provides insights into whole-body substrate oxidation, including glucose, fat, and protein. Participants are required to remain still, but not sleep, beneath the ventilated hood system for ∼15 min, after which the REE is calculated. Substrate oxidation is calculated as previously described [97].

### Data collection and management

Researchers involved in the study have received expert training in conducting PANSS, YMRS, and IDS-C interviews, performing electrophysiological measurements, administering cognitive tests, applying sensors, and collecting blood and saliva samples. Each participant is assigned a unique code for data storage, handling, and analysis, which cannot be directly traced back to the individual. Access to the identification key is restricted to authorized personnel. Data collection forms are completed on paper and subsequently entered into the eCRF (Castor EDC). To ensure data quality, assessors are thoroughly trained in Good Clinical Practice. Paper forms, including signed informed consent documents, are securely stored in a locked filing cabinet. Digital data are saved in a secured folder with restricted access for research personnel. Data will be retained for 15 y following the study's conclusion. Anonymous, selected material and data are shared after study conclusion with collaborators at the University of Texas (Department of Bioengineering, Dallas, USA) and the University of Alberta (Department of Pediatric Cardiology, Edmonton, Alberta, Canada) through Material and Data Transfer Agreements. Anonymous EnLiSense sweat sensor data are shared with EnLiSense LLC through a Data Transfer Agreement. All shared data and samples remain coded to protect participant confidentiality. An independent monitor performs quality assurance.

### Data analysis

#### Sample size

This study is the first to investigate the effects of KE in patients with SSD and BD and is designed as a pilot and feasibility study, precluding a formal sample size calculation based on pre-existing data. Because of the lack of consensus on sample size calculation in pilot/feasibility studies, we have relied on existing literature as a basis for our decision. Following Julious' recommendations [98], we have chosen a sample size of 12 subjects per group. Therefore, a total of 24 patients with either SSD (n = 12) or BD (n = 12) will be included in the study. If a participant drops out, efforts will be made to include a replacement.

### *Statistical analysis*

Comparisons regarding all outcome measures within patients (dGK condition compared with control drink condition) are performed using paired-samples *t* tests for normally distributed data (or Wilcoxon signed-rank test if parametric assumptions are not met). Group-level effects will be analyzed using a linear mixed effects model, with adjustment for confounding factors. A significance level of ≤0.05 will be used. Given the exploratory nature of this pilot study, no imputation analysis will be conducted in the case of missing data (except for missing EEG channels) and no correction for multiple testing will be applied.

### Data monitoring

#### Patient safety

This study utilizes well-established nutritional drinks with no known harmful side effects. Patients are clinically admitted to the department of psychiatry and are closely monitored by both the investigators and the clinical staff responsible for their treatment. In the highly unlikely event of an unexpected adverse effect, the procedures outlined in the study protocol will be followed. In accordance with Section 10, Subsection 4, of the WMO, the sponsor will suspend the study if there are sufficient grounds to believe that continuing the study may endanger the health or safety of the subjects.

#### Public disclosure and publication policy

Results from the study will be presented at patient information events (e.g., hosted by patient organization Anoiksis, members of which contributed to the design of this study). Additionally, manuscripts about the obtained results (regardless of whether the results are positive, negative, or neutral) will be submitted for publication in peer-reviewed international journals.

## Discussion

Our current study investigates the effects of KE compared with an isocaloric carbohydrate control drink in patients with SSD and BD. To the best of our knowledge, this is the first study to examine the impact of KE in this patient population. Although a few uncontrolled studies suggest beneficial effects of a KD on SSD and BD, dietary interventions can be challenging to study in a controlled setting [44,46,47,95,99,100]. In the context of SSD or BD, adhering to a KD poses an additional challenge, as core symptoms associated with cognitive deficits and negative symptoms like loss of motivation may decrease compliance. The challenging nature of a KD can also lead to selection bias, favoring patients with a lower disease burden and strong social support, thereby limiting generalizability. Importantly, during the acute disease phase, adherence to a strict diet is not feasible. Moreover, it takes several days to weeks to reach sufficient levels of ketosis after starting the KD, rendering this intervention unsuitable to achieve fast ketosis and thus unsuitable as a treatment strategy in the acute disease phase. Finally, psychiatric medications can inhibit ketogenesis, making it hard for patients to reach ketosis on a KD [101]. KE offers an interesting alternative, yielding acute, titratable ketosis. Studies using KE can, therefore, facilitate a more rigorous investigation of the effects of ketone bodies in SSD or BD, while also addressing remaining questions, such as whether achieving ketosis—and at what level—is necessary for symptomatic improvement on a KD. Additionally, if effective, they could provide a viable therapeutic strategy for patients with SSD or BD who struggle with dietary adherence, thereby making ketosis-based interventions more accessible to a diverse patient population.

Our study has several strengths. We consider the broad range of outcomes a strength, because it may inform future trials. Ketone bodies are hypothesized to improve symptoms of SSD/BD through multiple mechanisms. In this pilot study, we focus primarily on their effects on frequently observed electrophysiological abnormalities in SSD and BD (i.e., impaired PPI [53,54], resting-state EEG changes [[73], [74], [75], [76], [77]], and reduced P3B amplitude [73,78,79]). By assessing these electrophysiological outcomes, alongside perceived mood, energy levels, cognitive function and metabolic, oxidative stress, and immune parameters, our current pilot study seeks to understand how ketones may influence brain function in patients with SSD and BD. Ketone bodies may also improve symptoms of SSD or BD through less explored mechanisms, such as the circadian rhythm. Although a single administration of KE may not impact the circadian rhythm, continuously monitoring cortisol and melatonin levels in this study could provide deeper insights into the circadian rhythms of patients with SSD and BD.

Importantly, previous studies often lack consistent measurement of blood ketone levels. In this study, we include sequential measurements of blood ketone levels, providing detailed insights into the dynamics of BHB and after KE ingestion. We hypothesize that the induced hyperketonemia may lead to effects similar to those observed during endogenous ketosis –when ketone bodies are produced by the body itself—based on previous research demonstrating a metabolic competition between ketone bodies and glucose, favoring ketone utilization even in the context of an unchanged diet [27,57].

The randomized AB/BA crossover design of this study is expected to strengthen trial outcomes. In a crossover trial, participants function as their own controls, enhancing both the interpretability of results and the statistical power despite the limited sample size. Moreover, this design reduces interpatient variability in the comparison between groups and the effect of covariates such as participants’ demographic characteristics. Randomization to AB/BA can compensate for test–retest bias and potential carryover effects. By choosing electrophysiological outcome measures (relatively insensitive to test/retest effects), we further reduce bias in this crossover design.

A limitation of this pilot study is the use of a single ingestion of KE, instead of a longer period of supplementation. This limits the outcome measures to parameters with an expected acute response to ketosis. For instance, our current study cannot answer if negative symptoms would improve with KE. We aim to investigate this important question in future studies.

A known limitation of pilot studies investigating novel concepts is the lack of prior data, which prevents the ability to perform an informed power analysis. This, in turn, can weaken the resulting recommendations or conclusions. However, this gap in knowledge is precisely why our current study is necessary. It will provide the data needed to inform a subsequent randomized controlled trial, including a comprehensive power analysis. Another potential limitation is that do not assess whether the participants have a baseline deficit in PPI. It is known that not all patients with SSD and BD exhibit PPI deficits, which could limit the outcomes of our intervention. However, in this study, we will measure the percentage change in PPI after dGK ingestion compared with an isocaloric carbohydrate control drink, allowing each participant to serve as their own control thereby mitigating the effects of this limitation.

There are several biases that need to be considered in this study. The Department of Psychiatry at Amsterdam UMC functions as a regional hospital for acute psychiatric episodes, such as manic episodes or psychosis. The early-episode psychosis ward primarily admits patients who agree to their treatment and are willing to discontinue street drug use. This may introduce selection bias, as it represents only a subset of patients with SSD/BD. Conversely, including a more heterogeneous group could introduce additional confounders and affect the results. In addition, the substantial number of outcome measures in a moderately affected SSD/BD patient population presents a challenge, as it requires several (≤4) hours of intensive testing per test day. This could potentially lead to attrition biases, with more vulnerable patients dropping out of the study. However, we currently successfully finished data acquisition for 9 patients with SSD/BD in our study, without drop-out.

In conclusion, our current study is a first step in exploring the potential benefits of KE to improve symptoms in SSD and BD. By investigating the effects on electrophysiological outcomes, cognitive performance, metabolic, immune, oxidative stress, and circadian rhythm parameters, we aim to deepen our understanding of how ketones influence fundamental brain function related to behavioral and symptomatic manifestations in these patient groups. Our current study is a pilot study aimed at informing future larger-scale research with a longer period of supplementation and longer follow-up evaluating the potential of KE to improve symptoms in SSD and BD. If proven effective, KE could offer a novel and achievable therapeutic approach to modify pathophysiological mechanisms and thereby reduce functional impairments and alleviate the burden of these disorders.

## Author contributions

The authors’ responsibilities were as follows – KH, NJMvB, LdH, MRS: conceptualization; KH, YY, BO, DJAS, TZ, MJvT, SP, SM, MvdP, B-SR, RH, RWK: design & methodology; RLvdMvO, DAMD, DJAS, BO, MvdP: software; KH, DAMD, YY, RLvdMvO, DJAS, BO, MvdP: validation. AMK, DAMD: formal analysis; DAMD, RLvdMvO: investigation; DJAS, BO, LdH, SP, SM, MRS, JRBD: resources; DAMD, DJAS, BO, RLvdMvO: data curation; DAMD: writing - original draft; KH, ED, NvB, LdH, MJvT, BO, DJAS, MRS, AMK: writing – review & editing; KH, DAMD: visualization; KH, YY, MvdP, NJMvB, LdH: supervision; KH, MvdP, YY: project administration; KH, NJMvB, LdH: funding acquisition; KH, NJMvB, LdH: final content; and all authors: have read and approved the manuscript.

## Data availability

Data described in the manuscript, code book, and analytic code will be made available upon request, pending application and approval.

## Funding

This trial is funded by a Proof-of-Concept Grant (PoC) from Amsterdam Neuroscience, Amsterdam UMC (Project number: 28493), for the project titled “Ketones to Correct the Brain’s Bioenergetic Deficiency in Schizophrenia-Spectrum and Bipolar Disorders.”

## Conflict of interest

K. Huizer reports that equipment, drugs, or supplies and statistical analysis were provided by EnLiSense LLC. K. Huizer reports equipment, drugs, or supplies was provided by deltaG Ketones. The other authors report no conflicts of interest.
